# In Silico Clinical Trials in Drug Development: Virtual Patients, Applications, and Regulatory Convergence

**DOI:** 10.1002/cpt.70492

**Published:** 2026-09-27

**Authors:** Maximilian Balmus, Sheng‐Ya Wang, Edward W. G. Ashton, Rosie K. Barrows, Molly Monks, Yuzhang Ge, Arthur Lefebvre, Steven A. Niederer

**Affiliations:** ^1^ National Heart and Lung Institute, Faculty of Medicine Imperial College London London UK; ^2^ Division of Cardiovascular Medicine, Falk Cardiovascular Research Center Stanford School of Medicine Stanford CA USA

## Abstract

Conventional clinical trials remain the benchmark for evaluating therapeutic safety and efficacy, yet they are constrained by escalating costs, withdrawal over the extended follow‐up periods, recruitment difficulties, ethical limits, and a restricted ability to characterize heterogeneous populations. In silico clinical trials, which use computational models of patient physiology to simulate the effect of interventions across a virtual cohort, have emerged as a complementary paradigm that is efficient, scalable, and mechanistically informed. However, the maturity of in silico clinical trial applications varies considerably between physiological systems. This review pursues three aims. First, we introduce a common taxonomy for virtual patients and in silico trials, spanning levels of model personalization, from fully synthetic populations through hybrid cohorts to patient‐specific digital twins, and levels of abstraction, from compartment‐based models to whole‐organ anatomically accurate models. Second, we survey applications across multiple organ systems, drawing on illustrative examples that expose markedly different degrees of modeling maturity, from comparatively established cardiac and hepatic safety and efficacy studies to areas where mechanistic models remain early in development. Third, we examine the regulatory landscape, tracing its evolution towards risk‐informed credibility assessment, and the recent harmonization of model‐informed drug development guidance. Although personalized modeling and regulatory pathways are evolving, they often do not converge within a unified framework. Their wider adoption will require robust evaluation against experimental and clinical evidence to demonstrate their predictive reliability.

Conventional clinical trials remain the benchmark for establishing the safety and therapeutic efficacy of pharmaceutical interventions. Despite their central role in drug development, these studies face numerous challenges: escalating costs, prolonged development timelines, late‐stage drug attrition, ethical considerations, difficulties in patient recruitment, and a limited ability to comprehensively evaluate heterogeneous populations. Concurrently, advances in three areas: (1) mechanistic first‐principles modeling, including constitutive and physiological models^1^, (2) the automation in the analysis of clinical data, exemplified by convolutional neural network‐based segmentation^2^ and mesh generation tools,^3^, ^4^ and (3) model development tools^5^, ^6^, have significantly enhanced the accessibility and scalability of generating cohorts of synthetic or patient‐specific virtual models.

Against this backdrop, in silico clinical trials (ISCTs) based on multiscale and anatomically accurate models have emerged as a promising complementary paradigm for supporting the development of clinical interventions, whether pharmacological, device‐based, or surgical, through efficient, scalable, and mechanistically informed evaluation of therapeutic strategies.^7^ However, the field remains in its early stages, and its maturity is not uniform across treatment modalities or physiological systems. Device trials have benefited from a more mechanistically transparent device‐organ interface, whereas pharmacological applications, specifically when referring to models which integrate both quantitative system pharmacology (QSP) and multiscale anatomically accurate modeling, remain at an earlier stage of development. A similar asymmetry exists across physiological systems: Historically, research has concentrated on the cardiovascular system, yielding a broad range of models spanning varying levels of complexity and disease states, while other systems, such as the pulmonary system, have yet to reach comparable depth or breadth.

For these reasons, this review adopts a broad definition of ISCTs, encompassing research studies that use computational models to evaluate the effect of pharmacological therapies on virtual patients, ranging from fully synthetic virtual patient populations to patient‐specific representations. The virtual patient may be modeled at any level of abstraction, namely cellular, tissue, organ, or system.

The aims of this review are threefold. First, we introduce a common taxonomy for virtual patients and ISCTs, covering different levels of model personalization and abstraction. Second, we present a broad, though non‐exhaustive, survey of ISCT applications, illustrated through selected examples. Third, we discuss the current regulatory landscape surrounding ISCTs and consider future directions for their development and broader applicability.

## A TAXONOMY OF DIGITAL TWINS AND IN SILICO CLINICAL TRIALS

### Digital twins

Patient computational models are, in the most general sense, mathematical representations of a patient's physiology or disease that can be used to simulate how an individual responds to a drug or therapy. In this section, we expand on this concept and introduce an associated taxonomy, following the framework proposed by Pathmanathan et al.^8^

Models can range from simplified equation‐based descriptions of a biological process to detailed three‐dimensional anatomical reconstructions. Each model is built upon a baseline or template model: a predefined mathematical structure that captures the mechanism or system under study, along with relevant anatomical and physiological features, such as cardiac chamber geometry or lung airway topology. The behavior of each model is governed by a set of parameters—quantities that often map directly to physiological or physical properties, such as blood viscosity or the permeability of cellular ion channels.

In the context of patient‐specific applications, the parameter set can be divided into two subsets: fixed parameters, representing physical constants or population averages, and variable parameters, which can be adjusted to reflect individual characteristics. This distinction arises from both the incomplete datasets typically available for calibration and methodological limitations that constrain the number of parameters that can be estimated simultaneously. Historically, this classification relied on expert knowledge; more recently, however, methods such as global sensitivity analysis are increasingly used to provide a systematic and reproducible justification for tuning specific parameters in an individual patient.

Baseline models vary considerably in their spatial complexity and can be combined to represent multiple interacting physical systems simultaneously. One common classification criterion is the spatial dimensionality of the model:

- 0D models represent a system as a set of compartments with no spatial dimensions and are useful for capturing overall system behavior, such as the pressure–volume relationship of the heart;
- 1D models resolve a single spatial dimension and are commonly used to capture the physics of network‐based systems, such as blood flow through the arterial tree or airflow through the pulmonary airways;
- 2D models which represent the tissue as a shell, with common examples in atrial electrophysiology models^9^ and arterial wall models;^10^
- 3D models incorporate full anatomical geometry and enable the simulation of spatially varying phenomena, such as the propagation of action potentials through cardiac tissue, the distribution of mechanical stress through soft tissue, or drug transport and deposition throughout the body;

see **Table** **1** for a more comprehensive summary.

**Table 1:** Model dimensionality taxonomy: representation, assumptions, illustrative examples and computational cost

	System representation	Principal assumptions	Illustrative examples	Computational cost
0D	Connected compartments to representing organs and system	Spatial homogeneity and instantaneous mixing within compartments	Lumped‐parameter models of circulation; PKPD; QSP; action potential models	Seconds to minutes per simulation
1D	One resolved spatial coordinate along a connected network	Cross‐sectional averaging, predominantly axial transport	Lower‐airway transport in the whole‐lung deposition model; reconstructed renal vascular tree	Minutes, scales up with tree complexity, number of generations
2D	Tissue as a surface; fields defined over that surface	Negligible transmural variations	Atrial electrophysiology shell models	Intermediate
3D	Full anatomical geometry; state variables resolved as 3D fields	Constitutive laws for tissue behavior; boundary conditions	3D ventricular models; upper‐airway deposition CFD; lobule‐resolved liver twin	Hours per simulation on high‐performance computing

A summary of the principal assumptions, illustrative examples discussed throughout this review, and typical representative computational cost of each. At the model selection stage of an ISCT design process, the model fidelity required by a given context of use must be weighed against the computational cost it incurs and against the patient data required to construct or personalize it.

Models can also be classified along the related axes of multiscale and multi‐physics modeling. Multiscale modeling refers to the coupling of sub‐model representations operating at different spatial or temporal scales, a notable example being the linking of subcellular ion channel dynamics to tissue‐level electrical propagation and ventricular mechanics. Multi‐physics modeling, by contrast, refers to the coupling of distinct physical phenomena, such as lung tissue mechanics and airflow dynamics. As physical complexity increases, so do computational demands: Large 3D multiscale and multi‐physics simulations can require hours of runtime on high‐performance computing clusters.

In practice, 1D, 2D, and 3D models are constructed for a specific target organ and function and are coupled to lower‐dimensional representations of the remaining physiology. In the multiscale sense, for example, a 3D model of ventricular hemodynamics would be coupled to a 0D model of the systemic and pulmonary circulations to close the feedback loop. In the multi‐physics sense, distinct physical mechanisms may each be represented at different dimensionalities, such as 3D cardiac mechanics coupled to a 0D flow model.

Within this taxonomy, traditional pharmacokinetic (PK) and pharmacodynamic (PD) models fall within the 0D class, as do the lumped‐parameter circulation models and biophysical action potential models presented above, since dimensionality describes structural resolution rather than what a model represents. The two traditions differ instead in the direction in which a model is built,^11^ either top‐down or bottom‐up. PK/PD modeling is top‐down: It begins from the observed clinical response and describes the link between drug concentration and effect phenomenologically, through a function fitted to the observed exposure response rather than derived from the underlying biology. Mechanistic models are bottom‐up: They begin from cellular and/or physiological knowledge, allowing for the perturbation of system properties to observe changes in target output. Physiologically based pharmacokinetics is constructed in this second manner while predicting a pharmacokinetic endpoint and exemplifies that separation. The two directions are in practice combined more often than opposed: Ratano et al. combine pharmacokinetic and pharmacodynamic models of sedation with lumped‐parameter models of ventilation and acid–base homeostasis to predict the pressure‐based endpoints used to judge lung‐ and diaphragm‐protective strategies.^12^

Specifying a set of variable parameters on a baseline model produces a virtual patient, that is, a single realization of that model representing one plausible individual. Virtual patients can be divided into four groups based on the approach to parameter specification, each corresponding to an increasing degree of personalization, see **Figure** **1**.

**Figure 1:**
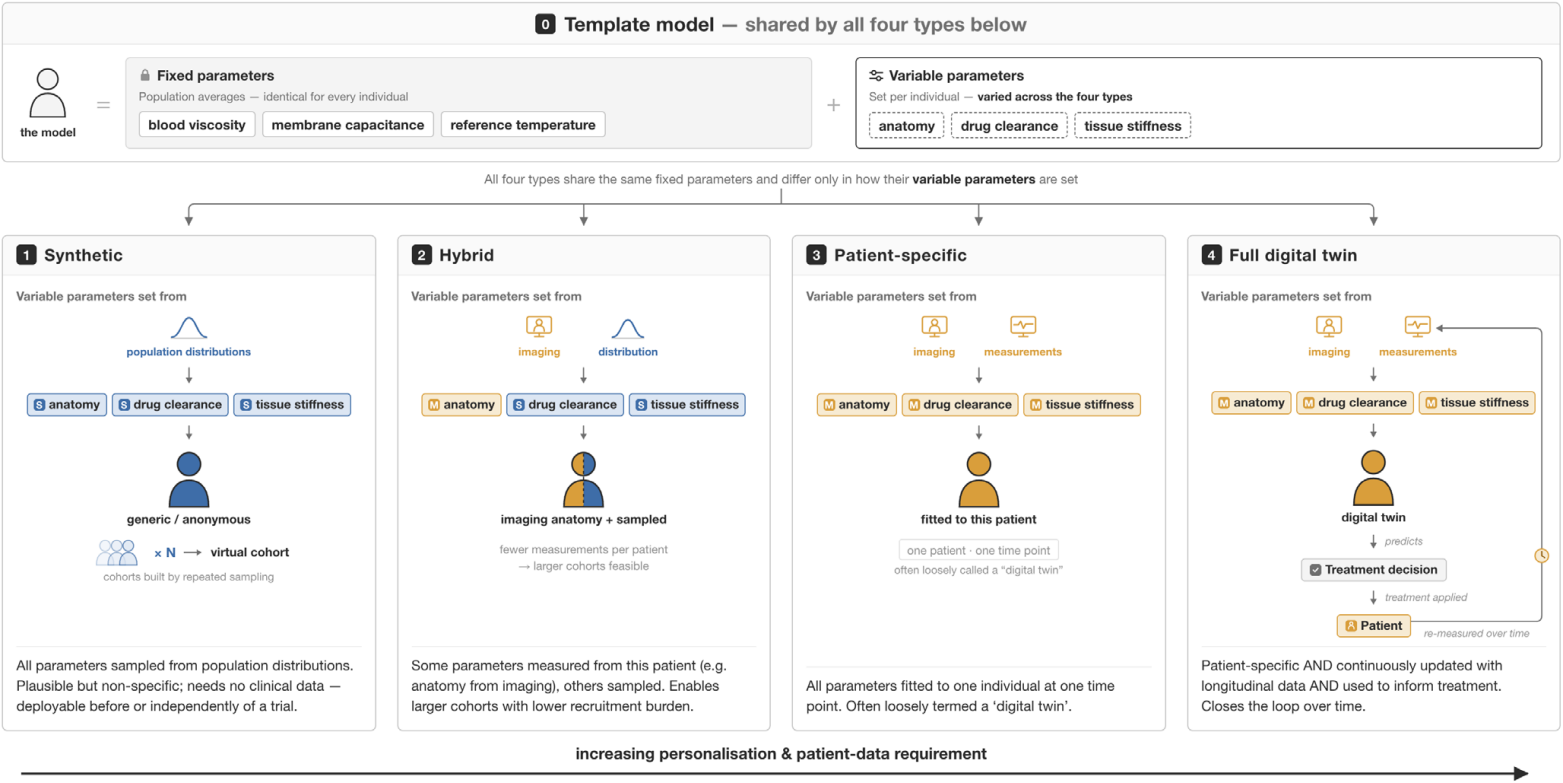
A taxonomy of virtual patients: one template model, four levels of personalization. All virtual patients derive from a shared template model whose parameters are partitioned into fixed parameters extracted from population‐average values, identical across individuals (illustrated by blood viscosity, membrane capacitance, and reference temperature) and variable parameters that are set per individual (illustrated by anatomy, drug clearance, and tissue stiffness). The four virtual patient types differ only in how these variable parameters are specified and are ordered by increasing personalization and patient‐data requirement. (1) Synthetic: all variable parameters are sampled from population distributions, giving plausible but non‐specific individuals that require no patient data and can be generated before, or independently of, a trial; synthetic cohorts are built by repeated sampling. (2) Hybrid: some parameters are measured or reconstructed from the individual (e.g., anatomy from imaging) while the other parameters are sampled, enabling larger cohorts with lower recruitment burden. (3) Patient‐specific: all variable parameters are fitted to a single individual at one time point only. This configuration is often loosely termed a “digital twin”. (4) Full digital twin: a patient‐specific model that is additionally updated continuously with longitudinal data and used to inform treatment, closing the loop as model predictions guide a treatment decision, the treatment is applied, and the patient is re‐measured over time. No published example yet satisfies all three requirements, so this category is shown as aspirational (dashed outline). Color encodes the source of each variable parameter: blue, sampled from a population distribution; gold, measured or reconstructed from the individual; split blue/gold, hybrid. A virtual cohort is a population of any of these types; an in silico clinical trial (ISCT) is a study performed on such a cohort. All parameters shown are illustrative.

Synthetic virtual patients are generated by sampling variable parameters from known statistical population distributions, producing plausible but non‐specific individuals. Hybrid virtual patients have some parameters sampled from population distributions (e.g., material parameters set from population averages) and others measured directly or reconstructed from patient data, such as cardiac geometry derived from medical imaging. Patient‐specific virtual patients are generated by fitting all variable parameters to data from a specific individual, producing a model calibrated to that patient.

In the literature, patient‐specific virtual patients are often referred to as digital twins (DTs). Strictly speaking, the term originates from engineering, and in a clinical context would denote a patient‐specific virtual model that is continuously updated using longitudinal patient data and used to inform treatment decisions.^13^ Such a capability could allow ISCTs to better explore time‐varying mechanisms, reduce model uncertainty, and enable adaptive trial designs. To our knowledge, no published example fully satisfying these criteria has been applied to ISCT. Throughout this review, we refer to the collection of virtual patients on which an ISCT is performed as a virtual cohort.

### In silico clinical trials

Unlike conventional clinical trials, ISCTs lack a formal classification and universally accepted definition, with terms such as “virtual trials”, “in silico studies”, and “in silico trials” used interchangeably across the literature. The use of the word “clinical” may itself be somewhat misleading: ISCTs generally do not fall within established regulatory frameworks and may involve patients that are partially or fully synthetic. In this sense, ISCTs elude standard classification, with concepts such as trial phases and control groups not mapping straightforwardly onto this emerging framework. More fundamentally, an ISCT does not reproduce the condition of real‐world use. Factors including adherence, dosing errors, comedications, contraindications, and sociological context fall outside the specified context of use of the studies surveyed here. We retain the term ISCT, which is established across the literature this review is aiming to consolidate,^8^, ^14^, ^15^ noting that the use of alternative terminology would further fragment the terminology. Within this broad category, the literature^16^ has begun to establish subtypes based on several considerations, which we summarize in the following:

The first is cohort construction, specifically, whether the cohort is composed of synthetic, personalized, or hybrid virtual patients. Synthetic cohorts carry the primary advantage of eliminating the need for clinical data during model construction and can therefore be deployed prior to, or independently of, a clinical trial. They may serve to provide early indications of treatment effectiveness, evaluate safety, or inform the design of subsequent trials. Personalized cohorts, by contrast, open the possibility of optimizing an intervention for individual subjects, for example, through dose titration. Hybrid cohorts offer a middle ground, allowing for inclusion of personalized virtual patients which can be supplemented with synthetic ones when recruitment is challenging.

The second consideration is whether the ISCT is cross‐sectional or longitudinal in design. The majority of published ISCTs fall into the former category, in part due to constraints on data availability. Virtual populations can also be distinguished as retrospective or prospective. Retrospective applications use existing clinical populations and observed outcomes to reproduce trial findings. These applications can compare responses to multiple treatments across patient subgroups and strengthen confidence in model predictions. By contrast, prospective virtual populations evaluate interventions before the relevant clinical outcomes are known, helping to assess therapeutic benefit and inform or refine trial design.

The third is the existence of an intervention model, that is, whether the action of the intervention is explicitly simulated, or whether its effect is instead captured through a snapshot approach. In the former case, a canonical example is modeling ion channel blockers by scaling the conductance of the corresponding channel by the fraction of unblocked channels, determined by the compound's IC50 and concentration.^17^, ^18^ In the latter, separate pre‐ and post‐intervention models are constructed to represent discrete snapshots of the target physiology using data from the patient both prior to and after the intervention is delivered to tune the patient model. The drug effect is then evaluated by comparing the model parameters between pre‐ and post‐intervention model parameters.

These considerations shape both the questions an ISCT can meaningfully address and the strength of the conclusions they can support.

## IN SILICO CLINICAL TRIALS: CASE STUDIES

In the following, we explore five major areas of ISCT applications: cardiac, pulmonary, renal, hepatic, and pancreatic diseases. Rather than being exhaustive in scope, the list is intended to be illustrative of the varying levels of modeling maturity, see **Figure** **2**, while also focusing on areas associated with substantial burden on healthcare systems around the world. Cardiovascular diseases remain the leading cause of death in the world, accounting for 1 in every 3 deaths, while chronic respiratory diseases, such as COPD and asthma, rank third.^19^ Hepatic diseases are responsible for about 2 million deaths annually (or 1 in 25 deaths worldwide).^20^ Chronic kidney diseases and diabetes, meanwhile, affect an estimated 788 million^21^ and 529 million^22^ individuals worldwide, respectively, with both conditions continuing to exhibit a rising global prevalence. In the sections that follow, each disease area is addressed in turn, with a brief introduction to the specific challenges identified within the literature, accompanied by a selection of illustrative examples.

**Figure 2:**
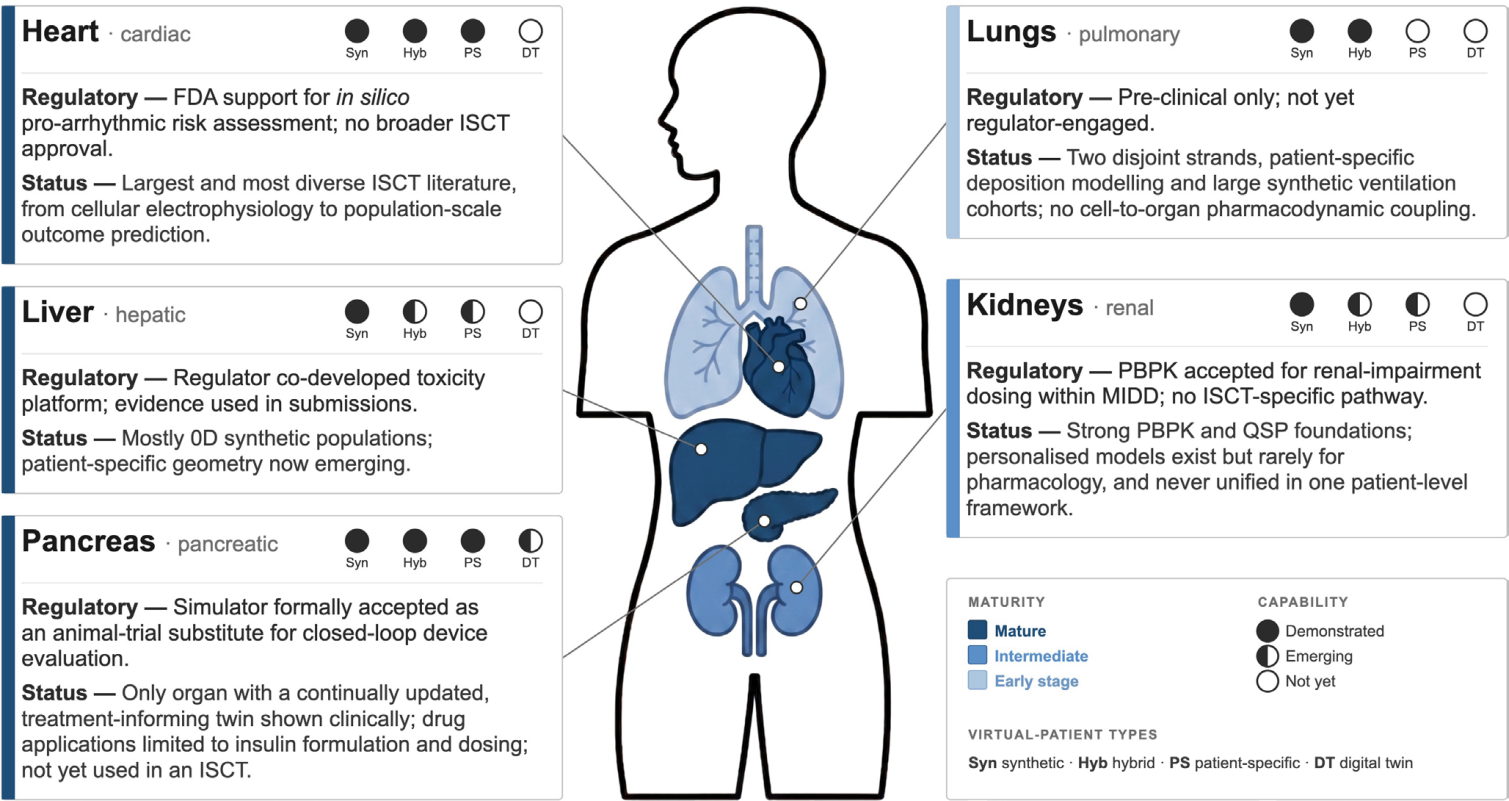
Maturity of in silico modeling across five organ systems. Each organ system is placed on a body map and summarized in a card scoring two independent channels. Fill color encodes maturity, a descriptor of regulatory and translational readiness rather than model sophistication, scored mature (virtual cohort evidence used in or endorsed by a regulatory process), intermediate (demonstrated ISCTs informing drug dosing or treatment decisions without an endorsed pathway), or early‐stage (models used only pre‐clinically). Capability records which classes of virtual patient (**Figure** **1**) have been realized: synthetic (Syn), hybrid (Hyb), patient‐specific (PS), and full digital twin (DT), each scored demonstrated (a peer‐reviewed application validated against independent data), emerging (proof of concept without external validation), or not yet (none identified in this review). The channels are independent, so systems sharing a capability profile may differ in maturity; scores throughout are the authors' assessment. The Regulatory line states each organ system's current regulatory engagement, while the Status line indicates its current state of the art. These engagements are named and cited in the corresponding case‐study sections; **Table** **2** maps at least one illustrative ISCT per organ system onto ICH M15 assessment elements.

### Cardiac applications

Cardiovascular drug therapeutics present a strong case for ISCTs because treatment responses are shaped by mechanistic interactions across cellular physiology, tissue mechanics and organ‐level function that conventional clinical trials cannot fully characterize. Virtual populations explicitly represent the variability in these underlying mechanisms. This allows therapeutic strategies to be evaluated under controlled conditions, addressing limitations where real‐world evidence is ethically constrained, costly, or difficult to obtain. Applications include pro‐arrhythmic risk assessment, generating rare or complex subgroups to expand trial cohorts and comparing long‐term outcomes across multiple therapies and patient populations.^23^, ^24^, ^25^, ^26^ By moving beyond trial designs that exclude patients and assume a shared phenotype, ISCTs can stratify patients by predicted efficacy and safety risk and prospectively inform studies to quantify uncertainty and increase confidence in predicted responses.^15^

Early prediction of cardiotoxicity is central to drug safety and efficacy assessment, yet animal and *in vitro* studies raise ethical concerns and do not always translate reliably to human responses.^26^ An early proof‐of‐concept applied a simple conductance‐block formulation to five existing rabbit, dog, and human ventricular‐myocyte base models. Mirams et al. (2011) imposed hERG, fast‐sodium, and L‐type calcium‐channel (IC_50_) values and therapeutic exposure ranges for 31 compounds and compared simulated biomarkers under hERG‐only and multichannel block.^18^ Clinical TdP‐risk categories were used to train and evaluate a linear discriminant classifier, but the underlying cellular models were not recalibrated for this context of use. FDA guidance identifies representation of the intended population through a virtual cohort and validation against population data as relevant evidence.^27^ The five fixed models demonstrate the mechanistic value of integrating multichannel effects but provide no evidence of cohort or population‐level variability for regulatory risk evaluation.

Pro‐arrhythmic safety is therefore a compelling use case for cardiovascular ISCTs, particularly within the FDA‐supported CiPA initiative, which promotes human mechanistic models for Torsade de Pointes (TdP) risk assessment.^28^ In a synthetic virtual population of 1,213 0D human ventricular action‐potential models with varied ionic conductances, Passini et al. (2017) simulated 62 ion channel‐interacting compounds using pore‐block drug models and varied ionic conductances to approximate intercellular variability.^26^ Model outputs of repolarization abnormalities and action potential biomarkers were validated against clinical reports. Repolarization abnormalities were predictive of clinical TdP risk with 89% accuracy, outperforming rabbit isolated‐heart experiments (75%). These controlled *in silico* conditions enable the systematic isolation of interacting potassium, sodium, and calcium‐current effects for multichannel drug assessment. Drug safety assessments were extended to coupled 0D electrophysiology and 3D ventricular tissue wedge simulations in a synthetic virtual population of 190 electromechanical models, testing 39 calcium‐channel blockers and CiPA compounds.^29^ This linked drug‐induced contractility changes to predicted ejection‐fraction variation, providing mechanistic insight into drug safety.

ISCTs can stratify patients by mechanistic differences represented across virtual patients to identify optimal therapies for individuals. This is relevant in atrial fibrillation (AF), where inter‐patient variability in ionic‐current properties and ECG metrics may explain therapeutic failure.^25^, ^30^ Dasí et al. (2022) embedded synthetic 0D atrial cardiomyocyte populations with varied ionic conductances into 3D whole‐atria electrophysiology simulations and exposed sustained AF to antiarrhythmic drugs.^25^ Cardioversion depended on prolonging refractoriness within specific ionic profiles, and logistic‐regression classifiers predicted drug response with 70% accuracy from ionic‐current and ECG features. This suggested that non‐invasive ECG may partially encode the ionic substrate and support stratification of AF treatment.^25^ A follow‐up hybrid virtual population incorporated structural remodeling, a major predictor of AF recurrence.^30^ Cardiomyocyte models were combined with patient‐derived low‐voltage area (LVA) distributions to generate virtual atria for 3D whole‐atria simulations across 12 drug scenarios. AF termination depended more on inward‐current phenotype than LVA extent alone.^25^ Together, these studies demonstrate how patient characteristics shape antiarrhythmic drug response. Direct human investigation is ethically challenging, as isolating mechanisms requires invasive and repeated drug testing within the same atrial substrate. Virtual populations, therefore, offer a tractable approach to mechanistic patient stratification and treatment selection.

ISCTs can address limitations in clinical trials arising from disease rarity and the long follow‐up periods needed to establish prognostic significance.^23^ Stine et al. (2025) show this in pulmonary arterial hypertension, a rare disease in which treatment responses vary with disease severity, time since diagnosis, and background therapy.^23^ They developed a 0D multiscale QSP model of the drug‐target signaling in endothelial and pulmonary arterial smooth muscle cells, controlling contractile forces and arterial remodeling. A synthetic 4,909‐patient virtual population was calibrated to five previous PAH clinical trials. Differences in disease severity across the five trials were reproduced by matching the proportion of virtual patients in each WHO functional‐class group to the proportions reported for each trial. The weighted population was then tested against a sixth longer follow‐up oral treprostinil trial (FREEDOM‐EV) with background therapy. The model predicted greater benefit from treprostinil in virtual patients with higher ET‐1 and lower PGI2 production, identifying a biomarker‐based patient subgroup whose selection increased the proportion of re‐simulated trials reaching statistically significant treatment effects.

Large sample sizes and long follow‐up are also challenges addressed by ISCTs to overcome in atherosclerotic cardiovascular disease modeling.^24^ The SIRIUS program aimed to predict, ahead of ongoing 5‐year clinical trials, whether lowering low‐density lipoprotein cholesterol (LDL‐C) with inclisiran, dosed only twice yearly, reduced long‐term risk of major adverse cardiovascular events (MACE) compared with placebo, ezetimibe, and evolocumab.^24^, ^31^, ^32^ The 0D mechanistic ASCVD model coupled lipoprotein homeostasis, plaque growth and rupture, risk‐factor effects, lipid‐lowering drug treatment mechanisms, with a Cox model‐based statistical survival model predicting cardiovascular death.^31^ A synthetic 204,691 virtual population was designed to reflect ASCVD patients with prior MACE.^32^ Relative to placebo, inclisiran was predicted to reduce LDL‐C by 49.7% and 5‐year 3‐MACE risk by 25.2%. SIRIUS illustrates how ISCTs can extend established lipid‐lowering evidence into long‐term outcome predictions, enabling prospective assessment of population‐ and subgroup‐level therapeutic benefit before conventional trial outcomes.

ISCTs are limited by the assumptions, simplifications, and data quality underlying the computational model and virtual population. Virtual cohorts only represent explicitly modeled variability and may omit relevant anatomical, physiological, demographic, or disease characteristics.^23^, ^25^ Simplified drug action and disease mechanisms also restrict clinical translation when pharmacokinetics and longer‐term adaptations are excluded.^29^ Computational cost further forces compromises between population size, model complexity, and simulation duration. More fundamentally, ISCTs primarily capture modeled biological and treatment variability, leaving real‐world factors such as non‐adherence, dosing errors, and unforeseen contraindications unrepresented. Consequently, ISCT predictions remain dependent on rigorous calibration and validation against experimental evidence and should be interpreted within their defined context to complement conventional clinical trials.^24^, ^31^

### Pulmonary applications

The lungs support gas exchange and consist of the airway tree, surrounded by a sponge‐like parenchyma. Chronic lung disease is an example where patient‐specific modeling can be used to account for variability in the patient population, as disease presentation is heterogeneous. However, in silico models of the entire organ, with a complete airway tree and vasculature and interaction with the nonlinear mechanics of the parenchymal tissue, are computationally expensive. Recently, two areas have emerged within lung modeling that use the ISCT framework: aerosolized drug use and mechanical ventilation protocols.

In drug deposition analysis, computational modeling is used to investigate the spatial distribution of drug delivery in the lungs, based on particle size and delivery device. When coupled with pharmacokinetic models of drug absorption, dissolution, and clearance, this approach allows for determination of the dose that is delivered locally to the desired region.^18^, ^33^ Previously, predictions of drug deposition had been limited to single population‐averaged geometries, but patient‐specific predictions are becoming more feasible. Grill et al. have recently developed a model capable of patient‐specific prediction.^34^ This model couples a 3D model of the upper airways with a 1D model of the lower airways. They completed validation with 6 patients and 10 inhalation experiments and reported good agreement and potential scalability. These models are currently used in the preclinical stages of drug development, for instance, for an inhaled anti‐CTGF/CCN2 protein.^35^ For this drug, simulations on one healthy and two IPF patients were completed to establish delivery of the drug in heterogeneous lung geometries.

ISCTs are also a viable approach when applied to mechanical ventilation, used to support patients in acute respiratory distress. In 2024, an ISCT was conducted to assess the benefits of lung‐ and diaphragm‐protective ventilation.^12^ This study used a virtual cohort of 5,000 synthetic patients simulating titration of ventilation and sedation protocols. A large virtual cohort was viable in this study as the reference models were lumped‐parameter models of ventilation, acid–base homeostasis, and the pharmacokinetic and pharmacodynamic effect of sedation. The ISCT was validated against outcomes from a previous clinical trial, showing reasonable agreement with an AUC of 0.83 with *n* = 30.

For in silico modeling to account for the mechanistic action of a wider variety of drugs and therapeutics, multiscale models of the lungs that can integrate cell‐level state changes to organ‐level function are required. A recent state‐of‐the‐art DT of ex vivo perfused lungs, developed by Zhou et al., incorporates multi‐modal data sets coupled to mechanistic simulations of airflow and perfusion.^36^ The model was validated on a simulated cohort of 50 simulated patients, and a separate cohort of 45 ex vivo perfused lungs. As this approach incorporates a broader range of biological data, it may offer an approach to test the effects of therapeutics, with an untreated DT acting as a counter‐factual to treated perfused lungs.

### Renal applications

The kidneys maintain fluid and electrolyte balance, regulate blood pressure, remove metabolic waste, and contribute to drug elimination. These functions make renal modeling relevant to chronic kidney disease (CKD), particularly diabetic kidney disease, as well as renal impairment dosing and drug‐induced kidney injury.

Renal modeling spans patient computational models, virtual cohorts, ISCTs, and DTs. Mechanistic kidney models are well established, while renal DT research is emerging across personalized prediction, anatomical reconstruction, and treatment‐oriented simulation.^37^, ^38^, ^39^ A clinical DT is commonly defined as a model calibrated to an individual, updated as new patient data become available, and used to inform treatment.^40^ The studies reviewed here contribute several of these components. PBPK also provides an established route for renal impairment assessment. Hsueh et al. modeled how reduced kidney function alters the pharmacokinetics of drugs cleared by renal organic anion transporters, illustrating its use in exposure prediction and dose selection.^41^

One prominent application is diabetes‐related kidney disease. QSP models extend this application by linking physiological regulation to treatment response. Hallow^42^ used a quantitative systems physiology model of renal function and blood pressure regulation to investigate possible renal and cardiovascular protection mechanisms of SGLT2 inhibitors.^43^ Saito and Nakada^44^ generated virtual patients with diabetic nephropathy and compared hyperkalemia risk across mineralocorticoid receptor antagonist scenarios. This is a direct example of treatment comparison within a renal virtual population. At the nephron scale, Hu and Layton^45^ extended a model of epithelial solute and water transport along a human nephron^45^, ^46^ to diabetes and SGLT2 inhibition, including changes in glomerular filtration and segment‐specific sodium and glucose transport. These models resolve treatment‐sensitive processes that are represented only in aggregate in whole‐system models.

Renal safety is another established application, particularly the prediction of drug‐induced kidney injury. Maass et al.^47^ coupled a proximal tubule microphysiological system with population PBPK and a QSP biomarker model. In vitro KIM‐1 responses to cisplatin, rifampicin, and gentamicin were translated into predicted plasma and urinary KIM‐1 profiles in 100 virtual patients. RENAsym extends this approach into quantitative systems toxicology by combining mechanistic kidney injury models with simulated populations to assess toxicity under therapeutic and overdose exposures.^48^ These platforms support population‐based comparisons of dose, treatment, and safety.

Taken together, renal modeling has strong foundations in mechanistic simulation and model‐informed drug development. PBPK supports exposure and dose decisions, while QSP and quantitative systems toxicology enable treatment and safety comparisons across virtual populations. Although patient‐specific renal models exist, notably for individualized risk prediction,^37^ alongside hybrid models using patient‐specific geometry for procedure planning,^38^ these personalized models remain rare in pharmacological applications. Integrating these capabilities with longitudinal clinical and imaging data could support renal DTs tailored to defined clinical decisions, with predictions evaluated against observed outcomes.

### Hepatic applications

Clinical trials to develop treatment for liver conditions such as hepatitis B (HBV) are often costly and resource intensive, creating a significant barrier to drug development,^49^ with drug‐induced liver injury (DILI), a form of hepatotoxicity, a major cause of clinical study termination and market withdrawal.^50^

Although liver ISCTs are not yet clinically ready, two decades of collaboration between academia, industry, and regulatory bodies show promise in virtual population and patient‐specific modeling, exemplified here by DILI assessment and chronic HBV drug development.

DILIsym is a mechanistic zero‐dimensional (0D) Quantitative Systems Toxicology (QST) model of DILI, developed through the DILI‐sim Initiative, a partnership of industry, academia, and the FDA.^51^ Using a PBPK whole‐body scaffold alongside known physiological parameter ranges, virtual populations (SimPops) are generated capturing inter‐individual variability.^51^, ^52^, ^53^, ^54^

First published in 2012, the platform is still in active use, including groups independent to the consortium.^55^, ^56^, ^57^, ^58^ Woodhead et al.^55^ modeled four next‐generation calcitonin gene‐related peptide (CGRP) receptor antagonists (rimegepant, atogepant, ubrogepant, and zavegepant) after hepatotoxicity ended late‐stage development of the first‐generation compound telcagepant, despite established efficacy.^55^ DILIsym used a virtual population of *N* = 285, predicting DILI in telcagepant while all four next‐generation drugs showed reduced likelihood of hepatotoxicity, later supported by clinical trials with three reported accepted by the FDA without hepatotoxicity warnings. Zavegepant has since been approved by the FDA.^59^ Further to QST models, liver disease mechanisms for HBV treatment have been developed. Treatment typically targets a “functional cure,” whereby HBV surface antigen (HBsAg) is undetectable and HBV DNA is suppressed to negligible levels for at least 24 weeks post‐treatment, currently achieved in fewer than 10% of cases.^20^, ^49^, ^60^ Cortés‐Ríos et al. demonstrated a workflow for ISCT, mirroring real‐world protocols.^49^, ^61^ A mechanistic infection model was fitted to biomarker data from *N* = 386 patients collated from literature, creating both patient‐specific virtual patients and population distributions. Sampling from these distributions generated *N* = 100,000 plausible patients, from which physiologically infeasible sets were rejected (~12%) and the remainder calibrated to match HBsAg baseline distributions. The subsequent demonstration ISCT, performed on a virtual cohort of *N* = 32,000, evaluated HBsAg loss rate at end‐of‐treatment (48 weeks) and functional cure rate at 72 weeks, reproducing the inverse relationship between baseline HBsAg and treatment response reported in the reference clinical study, although HBsAg loss differed quantitatively.

While synthetic population models provide representative physiological values, they lack patient‐specific geometric representation. Malka‐Markovitz et al. presented a cell‐to‐whole‐organ, 3D framework, incorporating patient‐specific geometry to create a “Human Liver Virtual Twin” for DILI modeling.^62^ Complementing the PBPK approach, the multiscale model represents 100,000 lobules capturing metabolic behavior, in addition to patient‐specific portal vein anatomy for blood flow computational fluid dynamics (CFD), found consistent with published 4D MRI flow data and predicted hepatocyte damage from acetaminophen. Application to a synthetic population framework as described offers promise as an increased fidelity hybrid population approach for future liver ISCTs. Only when, in addition to patient‐specific geometry, physiological quantities are also calibrated to specific subjects can a trial be considered patient‐specific.

### Pancreatic applications

The pancreas plays an important role in blood glucose regulation through β‐cell production of insulin. Type 1 diabetes (T1D) is an autoimmune disease whereby damage to pancreatic β‐cells results in a reduction in insulin production. In 2025, there were estimated to be 9.5 million cases of T1D globally, an increase of 13% since 2021, highlighting a growing problem.^63^

High prevalence and technological progression of closed‐loop metabolic monitoring devices known as the “artificial pancreas” could enable targeted and personalized treatment.^64^ The UVA/Padova T1D simulator was first accepted by the FDA as a substitute for animal trials for pre‐clinical study of insulin device closed‐loop control strategy in 2008.^65^ The work by Kovatchev et al. generated a synthetic virtual population of *N* = 300 (100 adults, 100 adolescents and 100 children) by sampling model parameters from distributions describing the T1D population. The model couples compartment models for glucose kinetics, stomach metabolism, and the gut with an insulin subsystem model. In addition to biological modeling, device control was also captured with continuous glucose monitoring (CGM) sensor and insulin‐pump error. This system showed alignment between in silico and in vivo measurement and formed the groundwork in establishing closed‐loop control simulators across the subsequent two decades. Following the simulator's acceptance, the authors reported that six insulin closed‐loop control algorithms have advanced to clinical practice, with two granted clearance for clinical use in both the United States and Europe.^64^

In addition to control algorithm approval, the model has been incorporated into clinical studies for dosing pre‐clinical assessment. A study by Visentin et al.^66^ used the UVA/Padova simulator to steer dosing of inhaled insulin (Technosphere insulin) for control of postprandial glucose (PPG).^66^ A virtual cohort of *N* = 100 patients was used, allowing optimization of dosing strategy to reduce hypoglycemic events, while a more recent study by Diaz et al.^67^ used the UVA/Padova simulator to assess ultrarapid‐acting insulin formulations AT247 and a faster‐acting formulation of insulin aspart (Fiasp) under open‐ and closed‐loop control systems.^67^ An adult synthetic cohort of *N* = 100 was used, demonstrating the requirement for calibrated device parameters for significant therapeutic benefit. Clamp experiments were simulated, matching experimental data for both AT247 and faster‐acting insulin aspart; however, simulation inputs were derived themselves from clamp experiment data. The closed‐loop nature of the system has driven development towards a true, continually updating DT, although not yet applied to ISCT. A systematic review by Cappon et al. identified eight T1D models described as DTs in the literature, comparing mathematical model, twinning procedure and validation to assess maturity.^68^ The review described two studies using replay simulations to incorporate patient‐specific CGM, insulin, and meal data to personalize a model and simulate alternative therapies using *N* = 100 virtual subjects in the UVA/Padova simulator.^69^, ^70^ Kovatchev et al. also utilized replay simulations to optimize the insulin delivery parameters bi‐weekly in a randomized clinical trial of *N* = 72 individuals with T1D, assessing human‐machine co‐adaptation.^71^ Here, individuals given access to the DT were permitted to experiment and alter treatment strategies, ultimately improving the primary outcome of time‐in‐range and reduced glycated hemoglobin. DTs therefore have proven potential for personalized treatment, and with increasing availability of data for calibration and validation, patient‐specific datasets could allow realization of DT‐enabled ISCTs.

## THE REGULATORY LANDSCAPE OF ISCTS

The DTs and ISCTs discussed above, while providing promising insights, cannot move beyond their status as research tools without regulatory acceptance. The regulatory landscape for computational modeling in medicine has developed along two distinct trajectories: the use of patient‐specific DTs to guide clinical decisions, regulated under medical device and Software as a Medical Device (SaMD) frameworks, and the use of ISCTs to generate evidence in pharmaceutical drug development, governed by model‐informed drug development (MIDD) guidelines, see **Figure** **3**. The former has driven much of the work on verification, validation, and credibility, but demand for computational evidence in drug development has increased, prompting regulatory bodies to extend existing frameworks. In this section, we discuss the evolution of this regulatory landscape, initiatives driving progress, available frameworks for verification, validation, and credibility assessment, and current barriers to broader adoption in the pharmaceutical pathway.

**Figure 3:**
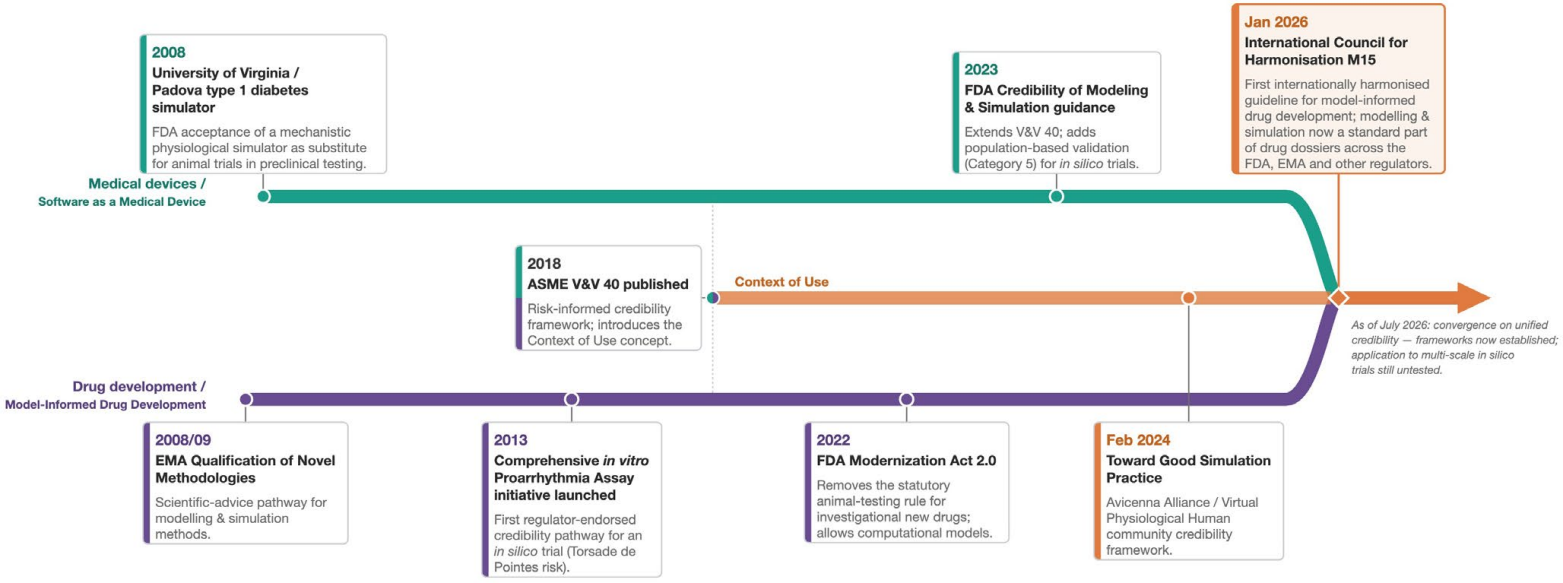
Evolution of the regulatory landscape for in silico medicine: two converging trajectories, 2008–2026. Regulatory acceptance of computational modeling in medicine has developed along two trajectories that are now converging. The device track (teal) covers patient‐specific digital twins used to guide clinical decisions, regulated under medical device and Software as a Medical Device (SaMD) frameworks; the drug track (purple) covers in silico clinical trials (ISCTs) generating evidence for drug development, governed by model‐informed drug development (MIDD) guidelines. The device track drove early work on verification, validation, and credibility, which the drug track later extended. Context of Use (CoU), introduced by ASME V&V 40 in 2018, forms the shared credibility backbone (orange) that both tracks adopt: Credibility must be established relative to a defined context of use. The trajectories converge with the community‐led Good Simulation Practice framework (2024) and, decisively, with International Council for Harmonisation (ICH) M15 (January 2026), the first internationally harmonized MIDD guideline, which makes modeling and simulation a standard component of drug‐development dossiers across the FDA, EMA, and other regulators. Circles mark milestones; the diamond marks convergence; the merged arrow denotes a unified credibility framework going forward. As of July 2026, these frameworks are established but their application to multiscale ISCTs remains untested. EMA, European Medicines Agency; FDA, U.S. Food and Drug Administration; V&V, verification and validation.

The use of modeling and simulation (M&S) evidence in drug development began with the EMA's Qualification of Novel Methodologies (QoNM) procedure, launched in 2008/2009, which allowed sponsors to seek scientific advice on innovative development methods including M&S.^72^ An important milestone came in 2018 with the publication of ASME V&V 40,^73^ which established a credibility assessment framework, informed by risk, and introduced the concept of Context of Use (COU)—the principle that model credibility is assessed relative to its specific intended use rather than in absolute terms. This framework, while initially intended for medical devices, has laid the groundwork for wider adoption. The CiPA initiative demonstrated that this was achievable in a pharmacological context, becoming the first community‐led and regulator‐endorsed credibility pathway for ISCTs of drug‐induced pro‐arrhythmia risk.^28^

The FDA Modernization Act 2.0 (2022) marked a turning point in legislation by removing the statutory requirement for animal testing in Investigational New Drug applications and formally permitting computational models as alternatives.^74^ The FDA subsequently operationalized credibility assessment for regulatory submissions through its 2023 Credibility of Computational Modeling and Simulation guidance,^27^ which extended V&V 40 and, with great relevance to ISCTs, introduced population‐based validation as a credibility category. Most recently, ICH M15,^75^ the first internationally harmonized guideline on model‐informed drug development, was adopted in January 2026 and establishes M&S as a standard component of drug development dossiers across FDA, EMA, and other ICH member agencies.

The FDA CMS Guidance and ICH M15 converge on a shared idea regarding credibility: Model credibility must be established relative to the specific COU. Both frameworks require sponsors to define the question of interest, the COU, the model influence, the consequence of an incorrect decision, and the resulting model risk. Both also stipulate that the rigor of verification and validation activities must be commensurate with this assessed model risk. In **Table** **2**, we provide a proposed mapping onto the key assessment elements of ICH M15 using examples the authors considered most aligned with the framework.

**Table 2:** Compilation of illustrative ISCT studies per organ, highlighting the study most closely aligned with ICH M15, alongside calibration and validation data

Application	Study	Calibration data	Validation data	Author assessment on M15 expectations
Cardiac	Angoulvant et al. (2024), Wang et al. (2025), Angoulvant et al. (2025)	Synthetic *N* = 204,691 population reflecting established ASCVD, capturing prior myocardial infarction, ischemic stroke or symptomatic peripheral arterial disease, residual LDL‐C elevation despite lipid‐lowering therapy, and inter‐patient variability in demographic and cardiovascular risk profiles. Other data: drug pharmacokinetics and treatment responses for statins, ezetimibe, evolocumab, and inclisiran; atherosclerotic plaque progression; and patient characteristics, lipid profiles, cardiovascular risk factors, and clinical outcomes from trials including ORION‐1, ORION‐10, and FOURIER.	ORION11: biological effect of inclisiran on lipoprotein levels ODYSSEY‐OUTCOMES: relation between reduction in lipid/lipoprotein levels under lipid‐lowering therapy (LLT) and reduction in CV event risk FOURIER‐OLE: CV outcomes over 5 years of follow‐up	Question of interest: Emulate the 5‐year efficacy of inclisiran vs. placebo, in combination with high‐intensity statin treatment, with or without ezetimibe, on (i) 3‐point MACE and (ii) cardiovascular death, in‐a virtual population with established ASCVD and LDL‐C ≥ 70 mg/dL. CoU: Predicting biological and clinical impacts of LLT in virtual patients with established ASCVD and LDL‐C ≥ 70 mg/dL at the population‐ and subgroup‐levels. Model Influence: Medium as of 28/08/26. No cardiovascular outcome data for inclisiran existed when SIRIUS was conducted, so the model outcome is the sole evidence for the predicted 25.2% reduction in 5‐year 3‐point MACE. However, inclisiran is already an approved LDL‐C lowering drug so no approval decision rests on this. Consequence of Wrong Decision: Low–Medium: Incorrect predictions could influence treatment considerations but are unlikely to cause direct harm because they complement an ongoing clinical trial rather than inform guidelines or clinical decisions. Model Risk: Reported as assessed, never shown. Model Impact: High: substantial departure from the randomized cardiovascular outcomes trial standard; no regulatory qualification or alignment reported
Pulmonary	Ratano et al. (2024)	*N* = 5,000, sampled from individual parameter distributions, which were determined elsewhere.	Comparison with in silico trial to pilot clinical trial with 30 patients (Dianti et al., 2022)	QoI: Can the model determine which ventilation procedure and sedation is required to achieve lung‐ and diaphragm‐protective targets? CoU: Not specified. In silicon trial results may be used to generate an Extracoporeal carbon dioxide removal score for use in clinical settings, although the authors state more data would be needed.
Renal	Vallejo et al. (2026)	*N* = 267 (simulated healthy adults, generated within the RENAsym platform; variability in oxidative stress sensitivity; no comorbidities). Data source: in vitro reactive oxygen species and oxygen consumption assays in HepG2 and renal proximal tubule epithelial cells; compound PBPK; injury and inflammation sub‐models calibrated on rodent cisplatin data from earlier RENAsym papers.	Compound level: three compounds withheld from the core cisplatin calibration (polymyxin B, valproate, acetaminophen), compared against published clinical outcomes; each compound's toxicity pathways were fitted to its own in vitro data. PBPK level: polymyxin B validated against five critically ill patients, four of five within the 0.5‐ to 2‐fold acceptance criterion; acetaminophen validated across oral and intravenous dosing.	QoI: Stated in substance, not in M15 terms – Does the platform correctly classify nephrotoxic potential for compounds not used in the initial calibration? CoU: Not defined. General intended use is given – to indicate kidney injury liability from in vitro inputs before clinical dosing.
Hepatic	Woodhead et al. (2022)	*N* = 285 virtual population (DILIsym SimPops calibrated in prior work). Data source: Parameter calibration against in vitro assays for reactive oxidative stress, mitochondrial dysfunction and bile acid transporter inhibition for all 5 compounds. Compound PBPK for telcagepant from literature, rimegepant from clinical trial data, zavegepant from animal data and in silico prediction, atogepant and ubrogepant from ADMET Predictor machine‐learning predictions in GastroPlus 9.5.	Telcagepant clinical alanine aminotransferase elevation data at 140 mg and 280 mg twice daily, 12 weeks dose protocol. At time of publication all four next‐generation compounds were corroborated by clinical trial data, with three approved by the FDA without hepatotoxicity warnings. Zavegepant since approved.	QoI: Implied from substance. Do next‐generation CGRP antagonists share telcagepant's hepatotoxicity? CoU: Not defined. General use of the model to assess hepatotoxicity risk within a drug compound class.
Pancreatic	Diaz et al. (2023)	*N* = 100 (adult cohort of the UVA/Padova T1D simulator calibrated in prior work – simulator accepted by FDA for substitute of animal testing in pre‐clinical evaluation of automated insulin delivery algorithms). Data source: UVA/Padova T1D simulator. Compound‐specific data and calibration from clamp experiments for AT247 and faster‐acting insulin aspart in 19 subjects.	No external validation dataset. Simulated insulin concentrations matched clamp experiments for AT247 and faster‐acting insulin aspart, but on the calibration dataset.	QoI: Implied from substance. Do faster insulin analogs benefit glycemia with tuned dosing and controller parameters? CoU: Not defined. FDA‐accepted simulator defining a therapy‐parameter design space.

M15 acronyms: QoI, Question of Interest; CoU, context of use; CoWD, consequence of wrong decision. Where CoU is not defined, all associated entries (Model Influence, CoWD, Model Risk, Model Impact) were removed as redundant.

ICH M15 sets out specific expectations for model evaluation. Verification activities should ensure that user‐generated codes are error‐free, that the equations reflecting modeling assumptions are correctly implemented, and that calculations are accurate. Validation and applicability assessment should demonstrate model robustness by evaluating the relevance of the data and model structure, the justification of assumptions, graphical and numerical diagnostics, and, where appropriate, external validation with independent data. The extent to which external validation is required is directed by the question of interest, the COU, and the model risk. While encouraged in all cases, external validation may be essential for higher‐risk applications.

The FDA CMS Guidance complements this by defining eight categories of credibility evidence, spanning code verification, model calibration, bench test and in vivo validation, emergent model behavior, model plausibility, and uncertainty quantification. Of particular relevance to ISCTs is Category 5: population‐based validation, which assesses credibility by comparing population‐level model predictions against clinical datasets rather than on a patient‐by‐patient basis.

While these two frameworks are generally aligned, they do diverge in certain specifics and in scope. ICH M15 introduces the concept of model impact, defined as the extent to which the proposed MIDD strategy departs from current regulatory standards or expectations. This is significant for ISCTs, where using a virtual cohort to address a question that would conventionally require a clinical trial constitutes, by definition, high model impact. In terms of scope, the FDA CMS Guidance acknowledges that its framework is expected to be applicable to ISCTs but stops short of providing specific recommendations for virtual cohort generation or ISCT execution, noting that best practices are still being developed. Contrastingly, ICH M15 explicitly encompasses the full range of M&S methods used in drug development, including quantitative systems pharmacology and toxicology, physiologically based pharmacokinetics, and disease progression models. This makes it directly applicable to the pharmacological applications discussed in this review.

These frameworks establish that computational evidence in drug development is no longer assessed ad hoc but through structured, risk‐informed credibility assessment. However, the practical application of these frameworks to multiscale mechanistic ISCTs remains largely untested. Model chains span cellular, tissue, and organ scales, each with distinct validation comparators and uncertainty structures, creating novel challenges for both regulators and modelers.

## CONCLUSIONS AND FUTURE DIRECTIONS

ISCTs are beginning to move beyond proof‐of‐concept, yet challenges remain. Computational expense remains non‐trivial for large‐scale 3D multiscale and multi‐physics simulations, constraining the size of virtual cohorts that can be generated within practical time frames. Data availability for true digital twinning, in the sense of continuously updated, patient‐specific models informing real‐time treatment decisions, remains limited outside a small number of experimental, well‐instrumented contexts, such as closed‐loop glucose control.^66^ Mechanistic modeling of several organ systems remains comparatively immature relative to the heart and, increasingly, the liver, limiting the disease areas in which high‐fidelity ISCTs can currently be deployed. Compounding this, in vivo, in vitro, and in silico research communities have historically operated with limited crosstalk, slowing the iterative calibration and validation cycles on which credible models depend.

On the regulatory side, no dedicated pathway yet exists for pharmaceutical ISCTs analogous to the device‐focused frameworks discussed earlier; sponsors must instead navigate model‐informed drug development guidance not originally designed with multiscale anatomical models in mind. This is compounded by variable reviewer expertise in mechanistic modeling, persistent gaps in the validation data needed to support population‐based credibility assessment, and the open question of how credibility should be apportioned across model chains spanning cellular, tissue, and organ scales, each with distinct uncertainty structures. Reproducibility and transparency of virtual cohort generation, legal and ethical ambiguities around the use of synthetic and hybrid patients, and the absence of lifecycle management guidance for models intended to update continuously together represent open regulatory questions rather than solved problems.

We anticipate progress concentrated where it has already begun, extending risk‐informed credibility frameworks such as ASME V&V 40 and the FDA Credibility of Computational Modeling and Simulation guidance to explicitly address multiscale, multi‐physics model chains, building on the precedent set by CiPA in cardiac safety. Community‐led, regulator‐endorsed initiatives analogous to CiPA in other disease areas would help establish accepted practice ahead of formal harmonized guidance. We also expect incremental standardization of virtual population generation and reporting.

Taken together, the trajectory of ISCTs mirrors that of computational evidence in drug development more broadly: from isolated research tools towards structured, risk‐informed components of the development pathway. Their wider adoption will depend less on any single technical advance than on the coordinated resolution of the technical and regulatory barriers outlined above, across a broader range of physiological systems and disease areas than has so far been demonstrated.

## FUNDING

SAN acknowledges support from the UK Engineering and Physical Sciences Research Council (EP/M012492/1, NS/A000049/1, and EP/P01268X/1), the British Heart Foundation (PG/15/91/31812, PG/13/37/30280, SP/18/6/33805), US National Institutes of Health (NIH R01‐HL152256), European Research Council (ERC PREDICT‐HF 864055), and the Alan Turing Institute. RKB received funding from the British Heart Foundation (RG/20/4/34803). MAB, RKB, YG and MM are funded by the MiMeC project, GSK (3000040406 / AGR2468). SW is supported by the National Heart and Lung Institute, Imperial College London and the UK Engineering and Physical Sciences Research Council (EP/W524323/1). EWGA is supported by the National Heart and Lung Institute, Imperial College London, through the Victor Phillip Dahdaleh Fellowship. AL acknowledges support from the UKRI AI Centre for Doctoral Training in Digital Healthcare (EP/Y030974/1). MM acknowledges support from EPSRC IDLA training grant (UKRI1983) and GSK (AGR3605).

## CONFLICT OF INTEREST

The authors declared no competing interests for this work.
